# Antimicrobial, Oxidant, Cytotoxic, and Eco-Safety Properties of Sol–Gel-Prepared Silica–Copper Nanocomposite Materials

**DOI:** 10.3390/ph18070976

**Published:** 2025-06-28

**Authors:** Lilia Yordanova, Lora Simeonova, Miroslav Metodiev, Albena Bachvarova-Nedelcheva, Yoanna Kostova, Stela Atanasova-Vladimirova, Elena Nenova, Iliana Ivanova, Lyubomira Yocheva, Elitsa Pavlova

**Affiliations:** 1 Faculty of Biology, Sofia University “St. Kliment Ohridski”, 8 Dragan Tsankov Blvd., 1164 Sofia, Bulgaria; lilijapj@uni-sofia.bg (L.Y.); nenova@uni-sofia.bg (E.N.); iaivanova@biofac.uni-sofia.bg (I.I.); 2 Department of Virology, Stephan Angeloff Institute of Microbiology, Bulgarian Academy of Sciences, 26 G. Bonchev Str., 1113 Sofia, Bulgaria; losimeonova@gmail.com (L.S.); metodiev.1996@abv.bg (M.M.); 3 Institute of General and Inorganic Chemistry, Bulgarian Academy of Sciences, 11 Acad. G. Bonchev Str., 1113 Sofia, Bulgaria; albenadb@svr.igic.bas.bg; 4 Institute of Metal Science, Equipment and Technologies with Hydro- and Aerodynamics Centre “Acad. A. Balevski”, Bulgarian Academy of Sciences, 67 Shipchenski Prohod Str., 1574 Sofia, Bulgaria; y_kostova@ims.bas.bg; 5 Institute of Physical Chemistry, Bulgarian Academy of Sciences, 11 Acad. G. Bonchev Str., 1113 Sofia, Bulgaria; statanasova@ipc.bas.bg; 6 Faculty of Medicine, Sofia University “St. Kliment Ohridski”, 1 Kozyak Str., 1407 Sofia, Bulgaria; lyubomirayocheva@abv.bg; 7 Faculty of Physics, Sofia University “St. Kliment Ohridski”, 5 James Boucher Blvd., 1164 Sofia, Bulgaria; 8 Center of Competence “Clean Technologies for Sustainable Environment—Water, Waste, Energy for Circular Economy”, 1000 Sofia, Bulgaria

**Keywords:** silica–copper nanocomposite, sol–gel method, antibacterial activity, ROS, cytotoxicity, eco-safety

## Abstract

**Background:** The present work is devoted to the biological effects of sol–gel-derived silica (Si)–copper (Cu) nanomaterials. **Methods and Results:** Tetraethyl orthosilane (TEOS) was used as a silica precursor; copper was introduced as a solution in ethanol with Cu(OH)_2_. The obtained samples were denoted as Si/Cu (gel) and Si/Cu/500 (500 °C heat-treated). Their phase formation and morphology were studied by XRD and SEM. The antibacterial activity was tested by two Gram-positive bacteria, three Gram-negative bacteria, and two types of eukaryotic species. Most bacteria were more sensitive to Si/Cu/500 materials than to Si/Cu (gel). The yeasts were more sensitive to Si/Cu (gel). The new nanomaterials were tested for oxidant activity at pH 7.4 (physiological) and pH 8.5 (optimal) in three model systems by the chemiluminescent method. They significantly inhibited the generation of free radicals and ROS. This result underlines their potential as regulators of the free radical processes in living systems. The epithelial tumor cell lines appeared more sensitive than the non-transformed fibroblasts, likely due to their metabolic activity and proliferation rates, leading to greater accumulation of the substances. Using *Daphnia magna*, the ecotoxicity study showed that the LC_50_ was reached at 1 mg/L of Si/Cu/500. Si/Cu (gel) was more toxic. **Conclusions:** Our results reveal the potential of these nanohybrids to be applied in living, eukaryotic systems. The cytotoxicity evaluation showed higher tolerance of normal, non-transformed cells, in concurrence with the oxidation tests.

## 1. Introduction

For ages, copper and its compounds have been recognized for their biocidal qualities. Copper is successfully used as an antibacterial agent in food and beverage packaging, as a wood conservation material in textiles and various paints, etc. Another major field of application is medicine, where copper-containing materials are used to minimize the spread of hospital bacterial infections. Copper compounds are also utilized as fungicides and bactericides to manage plant diseases in agriculture [1,2,3,4]. Nowadays, copper can also find many other applications in its nanoform. For instance, copper nanoparticle-functionalized thermoplastics are quite interesting because of their numerous applications in medical supplies, cleaning products, antiseptics, and many others. Several authors have reported the antibacterial effects of copper nanoparticles [5,6,7].

The preparation of nanoparticles requires a procedure that ensures a tight distribution of the particle size and controlled morphology. The sol–gel method provides better control to achieve these characteristics by using alcohols as solvents. Obtained by this method, the materials are classified as a new high-efficiency combination due to their organic and inorganic properties. Using this approach, it has been possible to obtain metal nanoparticles and metal oxides with a low distribution of size and controlled morphology [8,9]. Up to now, copper nanoparticles have been obtained by different techniques such as the molten state method, microwave-assisted, in situ, and sol–gel methods [4,10,11]. It is well known that a number of parameters affect the sol–gel process [12]. One of the important factors is the ratio of alkoxysilane precursors to water, as well as the pH of the reaction mixture [13]. The successful control of these parameters during the sol–gel process allowed us to obtain several kinds of materials with different physical properties and structures. To the best of our knowledge, the influence of the above-mentioned parameters is not yet clarified, which motivated our investigations in this direction and emphasized the novelty of the present work.

Silicon (Si)–copper (Cu)-based nanomaterials are an exciting class of hybrid nanocomposites that have gathered significant attention due to their remarkable properties and versatility, especially in the realms of medical and environmental applications. Si-Cu nanostructures combine the distinct advantages of both elements. Silicon is known for its biocompatibility, inertness, and low toxicity, while copper imparts superior antimicrobial properties and catalytic activity, making this combination particularly effective in various therapeutic and environmental applications [14]. One of their key advantages is their ease of synthesis, which often involves cost-effective chemistry methods that require minimal energy input. These methods can be scaled up, making these an attractive option for large-scale production [15,16].

The unique synergy between silicon and copper in these nanomaterials significantly enhances their antibacterial performance, making them a promising candidate in the fight against antibiotic-resistant pathogens [17]. As antibiotic resistance continues to rise, Si-Cu-based nanocombinations offer a potential solution by exhibiting potent antimicrobial properties that inhibit the growth and survival of harmful bacteria, thus providing an alternative or adjunct to traditional antibiotics. Furthermore, the oxidation activity of copper enhances their ability to generate reactive oxygen species (ROS), which can damage bacterial cell structures, further boosting their efficacy as antimicrobial agents [18]. This mechanism is particularly valuable in combating antibiotic-resistant bacteria, which have developed resistance mechanisms that make many conventional drugs ineffective.

When metal nanomaterials are used in living systems, they interact with cellular structures and metabolites. It is important to assess their safety or the opposite, their use as materials with antimicrobial and/or cytotoxic effects, for their application in various practical fields. Free radical reactions and the formation of ROS are vital metabolic processes that ensure the maintenance of homeostasis, the functional activity of the organism, and its adaptation. These processes are evaluated when assessing pharmaceutical substances, food additives, nanomaterials, and other impacts on the body [19,20,21]. Measuring the cytotoxicity of nanomaterials is essential to evaluate their safety for biomedical and environmental applications. Due to their unique physicochemical properties, nanoparticles may interact with cells unpredictably, potentially causing harmful effects.

Another compelling feature of Si-Cu nanoformulations is their low environmental impact. Their synthesis is often made using environmentally friendly techniques, and their components, particularly silicon, are abundant and naturally occurring, ensuring a minimal ecological footprint. Moreover, they have been shown to degrade in the environment without causing long-term harm, a significant advantage over more toxic or persistent nanomaterials [22]. In terms of future usage, Si-Cu-based nanocomposites hold great promise for various therapeutic applications, including drug delivery, wound healing, and biofilm disruption, in addition to their role as antibacterial agents. Their tunable properties allow for customization to meet specific needs, making them an attractive option for future therapeutic strategies [23].

In addition to the necessity of testing and describing all newly synthesized materials, it is also of great importance to describe materials at the nanoscale, since their properties often differ from what is expected and from those observed of the substances of chemical elements at the macroscale. The reduced size of nanomaterials leads to improved mechanical strength, electrical conductivity, and optical properties. Moreover, at the nanoscale, materials often also demonstrate increased chemical reactivity due to their high surface energy [24]. One should always take into account their mechanical effects on the living organism too.

In the present work, silica–copper hybrids containing metal nanoparticles were synthesized using the sol–gel approach. The antibacterial activity of Si/Cu (gel) and Si/Cu/500 (heat-treated at 500 °C) against two Gram-positive bacteria, three Gram-negative bacteria, and two eukaryotic species was subsequently examined. The minimum inhibitory concentration (MIC) and the minimum bactericidal concentration (MBC) were evaluated to estimate their microbicidal effects. Their oxidant activity against free radicals and ROS in three model chemical systems was tested, describing the mechanism of their biological effects. Cytotoxicity tests in two epithelial transformed cell lines and two fibroblast non-tumor cultures (human, canine, murine) were performed with determination of CC_50_ (50% cytotoxic concentration). Their ecological safety was tested by the most sensitive organism chosen as an indicator in freshwater international standards—the crustacean *Daphnia magna*. All these assays describe their possible applications and limitations for practical purposes. The novelty in this work is in its broad biological evaluation of the antibacterial activity of the sol–gel-synthesized Si-Cu nanomaterials beyond traditional antimicrobial testing. In addition to previous works, we have tested cytotoxicity in different cell lines, oxidative reactivity, and ecologic safety in *Daphnia magna*, resulting in an original multi-model study. Furthermore, the effect of the sol–gel fabrication conditions on the hybrids’ bioactivity is yet to be fully elucidated, but is also discussed. The synthesis method provides a well-defined approach to control nanoparticle properties and thus increases their applications for biological and environmental uses. This combined study presents valuable insights on the safe and efficient applications of hybrid Si-Cu nanomaterials.

## 2. Results and Discussion

### 2.1. XRD and SEM Observations

**Figure 1:**
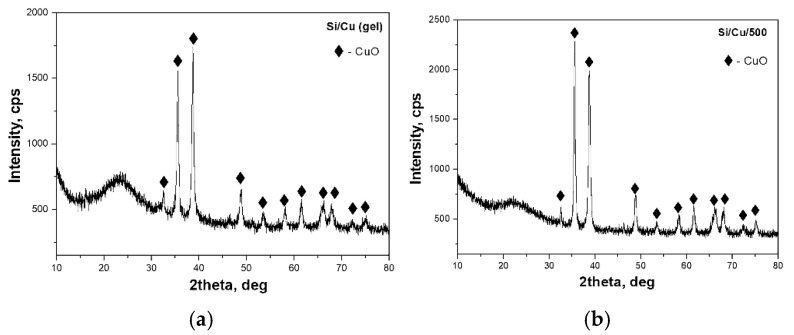
XRD patterns of the prepared Si/Cu (gel) hybrid (**a**) and Si/Cu/500, heat-treated at 500 °C (3 h) powder (**b**).

XRD analysis was used to verify the phase formation in the investigated samples. X-ray diffraction patterns of the prepared gel and heat-treated powders are shown in Figure 1. As presented, the amorphous halo is preserved in both samples along with the diffraction peaks typical for CuO, the three strongest lines at 2θ with 35.1°, 38.2°, and 48.6° (JCPDS 080-1916), which correspond to the reflection from the (002), (111), and (202) planes, respectively. The amorphous halo is a typical characteristic of Si-based nanomaterials obtained from TEOS as a precursor, and it is registered around 2θ ≈ 20° [25]. As seen in Figure 1, the presence of the typical diffraction peaks of CuO remains in the heat-treated sample (Figure 1b) as the intensity of the diffraction line (002) became stronger. Obviously, the thermal treatment increased the sample’s crystallinity, which leads to an increase in the number of grains oriented in a particular direction. It was already found that by increasing the annealing temperature, many different grains of the same orientation coalesce together and form a large grain having a certain orientation. Therefore, the diffracted intensity is supposed to increase as the crystallite size increases. This proves that texture may change during grain growth and affect the kinetics in the process of rapid annealing [26]. Moreover, new phases were not detected. The average crystallite size of the particles, calculated from the broadening of the diffraction line using Sherrer’s equation, is about 25–30 nm.

**Figure 2:**
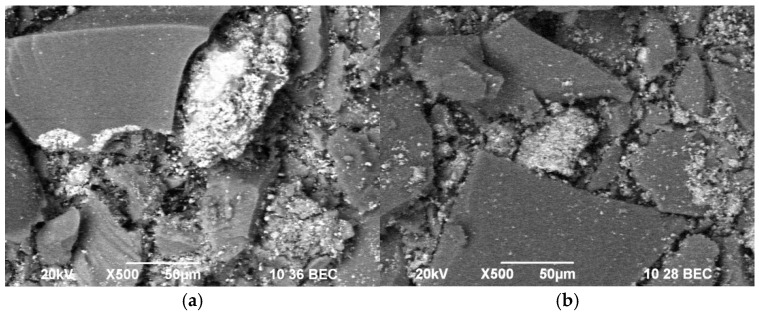
SEM images of the samples: Si/Cu (gel) (**a**) and Si/Cu/500, heat-treated at 500 °C (3 h) powder (**b**).

SEM observations of the samples are shown in Figure 2a,b. The electron microscope images demonstrate a plate-like surface texture with a tendency towards agglomeration. The size range of the observed particles varies between 15 and 80 nm, which is per the already reported data [27]. The surface of the heat-treated sample appeared rougher. The reason for this phenomenon could be the fast evaporation of TEOS and the occurrence of hydrolysis–condensation processes [28].

**Figure 3:**
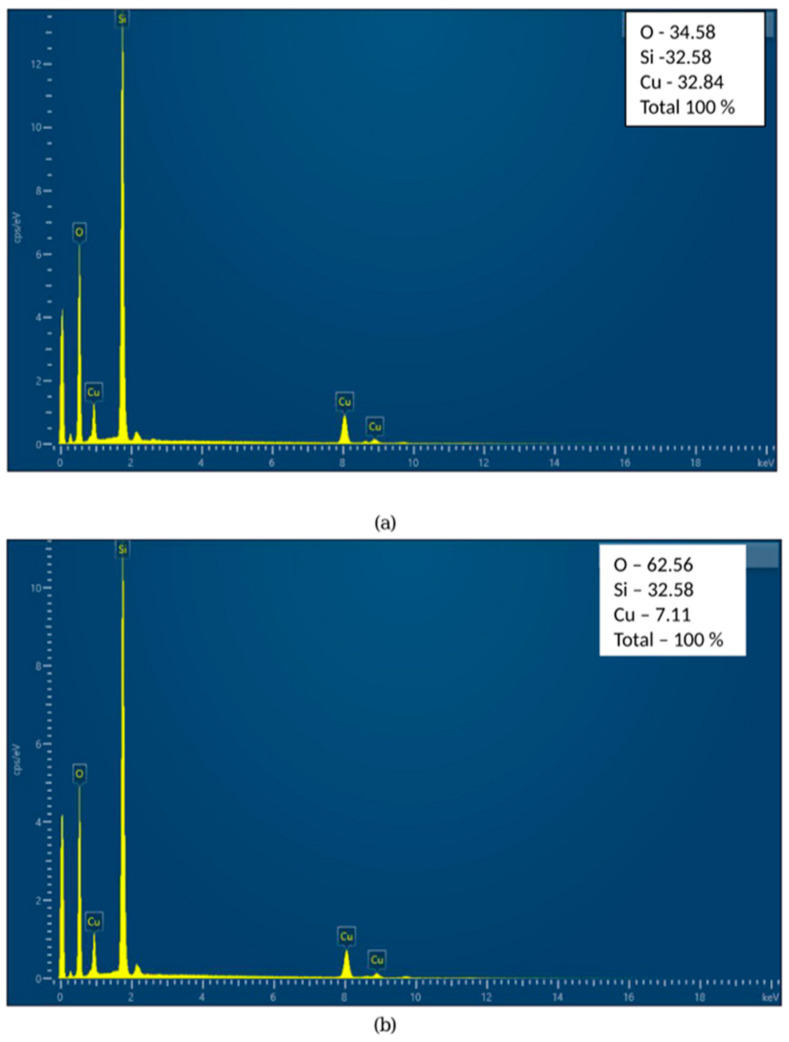
EDS analysis of the samples: Si/Cu (gel) (**a**), Si/Cu/500, heat-treated at 500 °C (3 h) powder (**b**).

EDS analysis revealed the presence of silica and copper components in both samples, as shown in Figure 3a,b. The increase in oxygen content and decrease in Cu content have already been found and explained by many authors [29,30]. During heating in air, copper readily oxidizes, which introduces oxygen atoms into the sample, and EDS analysis detected additional oxygen atoms, increasing the measured O percentage. Additionally, copper diffused into the amorphous Si matrix, which resulted in its redistribution and reduced the Cu surface concentration [31].

### 2.2. Antimicrobial Activity Testing

**Figure 4:**
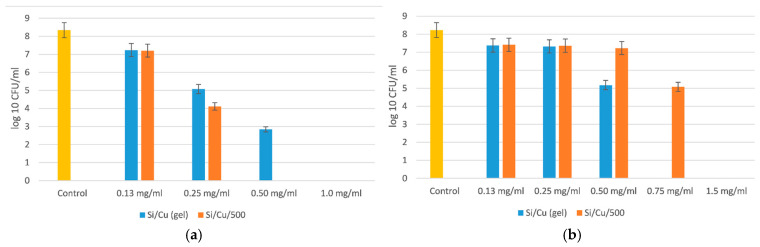
The effect of Si/Cu composites on *Salmonella enterica* ATCC 14028 (**a**) and *Escherichia coli* ATCC 25922 (**b**).

**Figure 5:**
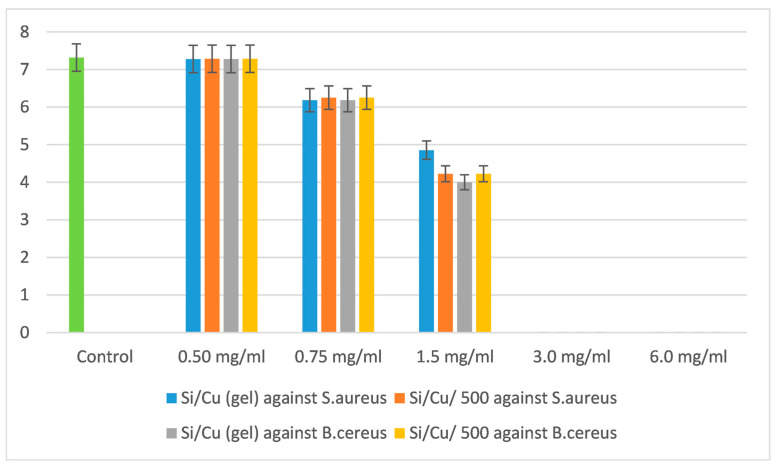
The effect of Si/Cu composites on *Staphylococcus aureus* ATCC 25923 and *Bacillus cereus* ATCC 11778.

**Figure 6:**
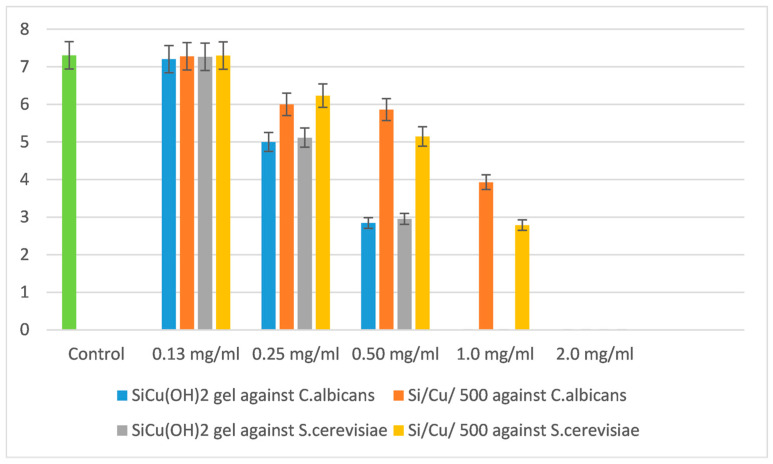
The effect of Si/Cu composites on *Candida albicans* ATCC 18804 and *Saccharomyces cerevisiae* CCY 21-6-3.

**Figure 7:**
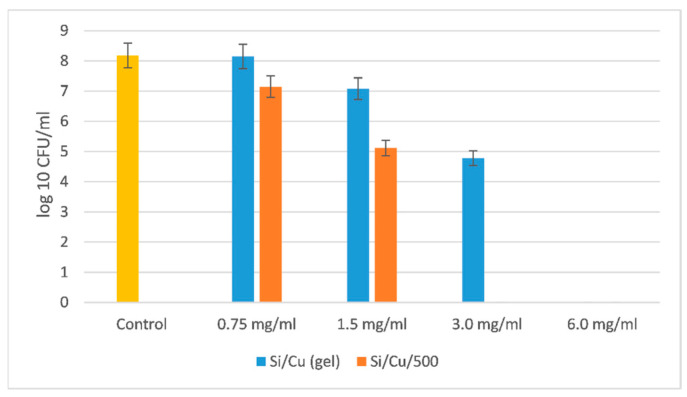
The effect of Si/Cu composites on *Pseudomonas aeruginosa* ATCC 27853.

The antimicrobial effect was tested from 0.13 to 6.0 mg/mL with different bacteria and yeasts and was proven to be species-specific. Those most sensitive to the newly synthesized material were *Salmonella enterica* ATCC 14028 and *Escherichia coli* ATCC 25922. Two Gram-positive bacteria, *Staphylococcus aureus* ATCC 25923 and *Bacillus cereus* ATCC 11778, had similar inhibitory and bactericidal concentrations, as did the yeasts *Candida albicans* ATCC 18804 and *Saccharomyces cerevisiae* CCY 21-6-3. The most resistant bacterium to the Si/Cu (gel) material was *Pseudomonas aeruginosa* ATCC 27853 (Figure 4, Figure 5, Figure 6 and Figure 7).

As seen from Figure 4, the minimal bactericidal concentration (MBC) of Si/Cu (gel) for *Salmonella enterica* ATCC 14028 was 1 mg/mL, and for the heat-treated hybrid nanomaterial it was 0.50 mg/mL. The species-specific activity of the new material was evidenced by the reduced effect of the heat-treated Si/Cu/500 hybrid on *Escherichia coli* ATCC 25922. The MBC of Si/Cu/500 was 1.5 mg/mL, and for Si/Cu (gel) it was 0.75 mg/mL. The results show the higher resistance of *Escherichia coli* ATCC 25922 in comparison with *Salmonella enterica* ATCC 14028.

The MBC of the newly synthesized materials on both bacteria was 3.0 mg/mL, and the minimal inhibitory concentration (MIC) was 1.5 mg/mL. Figure 5 presents the inhibition on the growth of both bacteria, observed as a 4-order of magnitude in comparison with the control variance.

Yeasts are usually more resistant than bacteria, but our results have shown similar sensitivity to the newly synthesized Si/Cu nanoparticles, as demonstrated in Figure 6.

Both tested yeasts were killed by concentrations of 2.0 mg/mL by the heat-treated Si/Cu/500, and 1.0 mg/mL by Si/Cu (gel). The MBC was the same. The MIC had about a 4-order-of-magnitude effect on the quantity of bacterial-treated cells in comparison with the control variance (Figure 6). Both yeasts were more sensitive to Si/Cu (gel) nanoparticles than the heat-treated material.

The most resistant microorganism was the Gram-negative bacterium *Pseudomonas aeruginosa* ATCC 27853 (Figure 7).

The MBC for *Pseudomonas aeruginosa* ATCC 27853 was two times higher than the one for Gram-positive bacteria *Staphylococcus aureus* ATCC 25923 and *Bacillus cereus* ATCC 11778; five times higher than the MBC for the yeasts *Candida albicans* ATCC 18804 and *Saccharomyces cerevisiae* CCY 21-6-3; and approximately five–six times higher for the Gram-negative bacteria *Salmonella enterica* ATCC 14028 and *Escherichia coli* ATCC 25922. It is very interesting to understand how that bacterium succeeded in resisting such a high concentration of nanoparticles. Some clues can be found in Pang et al. [32].

In conclusion, all bacteria were more sensitive to the gel Si/Cu materials than the heat-treated nanoparticles. Just the opposite effect was observed for the yeasts, which were more resistant to the gel nanoparticles and more sensitive to the heat-treated ones (Figure 6).

Bacteria possess a peptidoglycan cell wall structure, but that of yeasts is formed by chitine, proteins, and mannan [33]. Our results show that the gel hybrid material interacts better with the outer membrane and peptidoglycan layer of the bacterial envelope, but the material heated at 500 °C interacts more strongly with the mannan fibrils, proteins, and chitin from the cell wall of the yeasts.

All the results support the antimicrobial effect of both newly synthesized Si-Cu nanocomposites. The MBC was between 0.5 and 3.0 g/L, with the exception of *Pseudomonas aeruginosa* ATCC 27853 where the bactericidal effect was achieved at 6 g/L for the Si/Cu (gel) and 3 g/L for the Si/Cu 500.

Most known mechanisms of antibiotic resistance have little impact on nanomaterials, as nanoparticles exert their effects primarily through direct interaction with the bacterial envelope [34]. In addition, they form reactive oxygen and nitrogen species that destroy various macromolecules important for cellular functions. This proves that nanoparticles and nanoceramics will be less likely to induce resistance in bacteria than the widely applied antibiotics [35,36].

### 2.3. Chemiluminescent Oxidation Tests

The newly synthesized nanocomposites tested exhibit inhibition of the oxidation reactions across all model chemical systems designed for the generation of free radicals and ROS. This effect is likely attributed to the following:

- The relatively large particle size, which may promote agglomeration;
- Their high mass, limiting effective interaction with the added reagents despite the application of various stirring and homogenization procedures;
- The exceptionally stable incorporation of the copper ions (Cu^2+^) within the matrix, preventing their active participation in the model free radical and ROS-generating reactions, including the Fenton reaction.

Despite the native high reactivity of copper and the expected release of Cu^2+^ ions, which would normally be anticipated to intensify the observed oxidation reactions, the recorded chemiluminescent signal was consistently lower than that from control reactions without the participation of nanomaterials. The observed effect can be characterized as inhibitory toward free radical-mediated oxidation reactions, rather than antioxidant, due to the electronic structure of silicon and copper within the matrix. These findings suggest that the newly synthesized Si/Cu hybrids could serve as controlling agents within living systems or organisms with regard to the free radical generation and oxidation reactions.

The experimentally confirmed safety and biocompatibility of the materials would enable their application in the following practical areas:

- As carriers for drug delivery systems, allowing controlled release within the organism;
- Incorporation into biomaterials used for implants or artificial tissues, where prevention of oxidative stress and cellular damage is critical; the newly synthesized hybrid materials exhibit enhanced properties and demonstrate synergistic effects between silicon and copper compounds, including improved electrical conductivity, thermal stability, and mechanical strength;
- At low concentrations, application in products requiring antibacterial, yet non-cytotoxic, surface properties.

**Figure 8:**
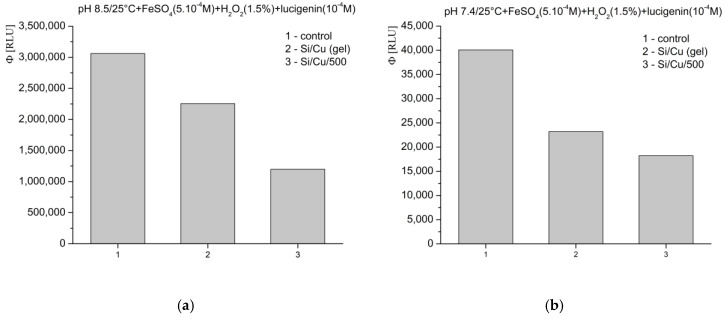
Effect of the newly synthesized nanomaterials on chemiluminescence in a system for the generation of ·OH and ·OOH radicals, at pH 8.5 (**a**) and pH 7.4 (**b**), presented as quantum yields.

In Fenton’s system for the generation of ·OH and ·OOH radicals, at pH 8.5—optimal for radical formation—the tested materials exhibited an inhibitory effect, as follows: Si/Cu (gel) suppressed luminescence by more than 26%, and Si/Cu/500 by more than 60% (Figure 8a). When the same reaction was measured at pH 7.4—corresponding to the physiological conditions for bacteria and the internal fluid environment of the human organism—similar effects were observed. The materials effectively inhibited the signal: Si/Cu (gel) by approximately 42% and Si/Cu/500 by approximately 55% (Figure 8b).

**Figure 9:**
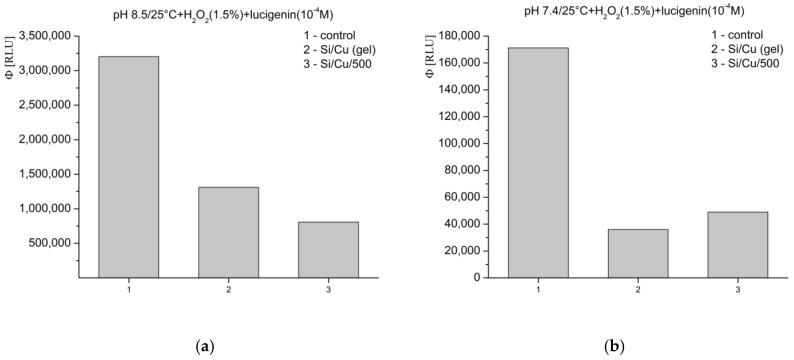
Effect of the newly synthesized nanomaterials on chemiluminescence with oxidant H_2_O_2_, at pH 8.5 (**a**) and pH 7.4 (**b**), presented as quantum yields.

In the model system where H_2_O_2_ acts as both a strong applied oxidant and an ROS, the registered inhibition effects at pH 8.5 were as follows: Si/Cu (gel) suppressed the signal by nearly 60%, while Si/Cu/500 suppressed the luminescence by almost 75% (Figure 9a). At pH 7.4, the nanohybrids showed the following suppression: Si/Cu/500—over 70%, and Si/Cu (gel)—nearly 80% (Figure 9b).

The Fenton’s and the H_2_O_2_ oxidation systems are indicative of whether copper ions (Cu^2+^) would be released from the matrix and thus enhance the chemiluminescent signal above the level observed in the control reaction (without the participation of nanoparticles). In both model chemical systems, copper ions could, theoretically, interact with H_2_O_2_. However, they are neither strong chemical competitors to the iron ions (Fe^2+^) nor do they appear to be spatially or thermodynamically competitive, despite the relatively high applied concentrations of each tested Si/Cu hybrid nanomaterial (1 mg/mL).

**Figure 10:**
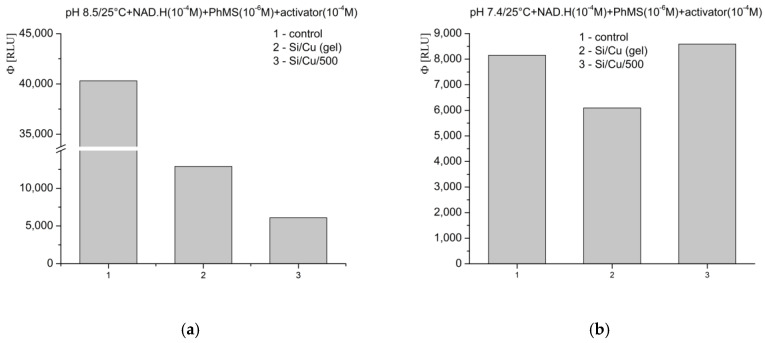
Effect of the newly synthesized nanomaterials on chemiluminescence in a system for the generation of O_2_^−^··radicals at pH 8.5 (**a**) and pH 7.4 (**b**), presented as quantum yields.

In the third investigated model system, at pH 8.5—a slightly alkaline environment favoring strong superoxide (O_2_^−^·) radical generation—the tested newly synthesized nanoformulations strongly suppressed light emission, as follows: Si/Cu (gel) suppressed luminescence less, at nearly 70%, while Si/Cu/500 achieved almost 85% (Figure 10a). At pH 7.4, under physiological conditions, the tested nanocomposites exhibited a weak inhibitory effect, with registered quantum yields of the reactions close to those of the control (Figure 10b). The obtained results were as follows: Si/Cu (gel) inhibited the signal by over 25%, whereas Si/Cu/500 demonstrated approximately 5% pro-oxidant activity.

In conclusion, the obtained results from the luminescent assay clearly show that the investigated nanomaterials exhibit a significant inhibitory effect on the generation of ROS in various tested model chemical systems. This effect is highly influenced by the pH level. At physiological pH 7.4, the effects varied between the different materials, with Si/Cu/500 even exhibiting extremely weak pro-oxidant activity. This highlights the importance of carefully selecting nanostructures depending on the specific medium and intended application, while also demonstrating the potential of the studied nanohybrids as regulators/inhibitors of free radical processes in the living organism.

### 2.4. Cytotoxicity Tests

**Figure 11:**
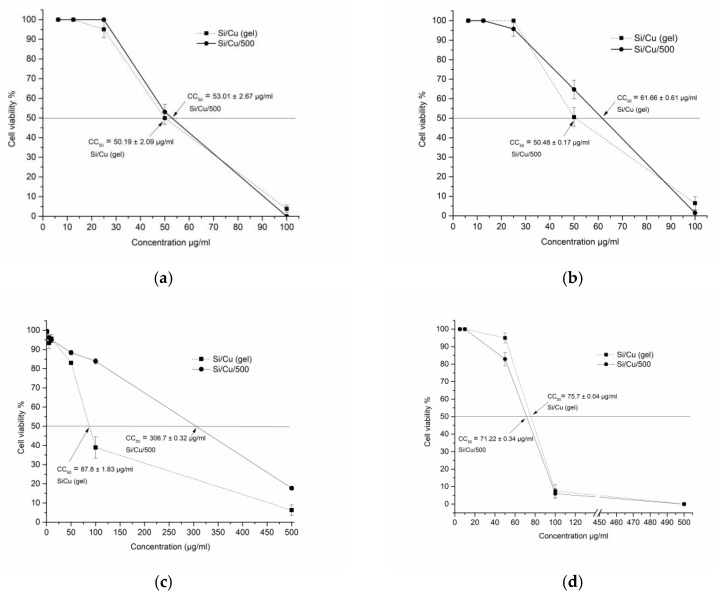
Determination of 50% cytotoxic concentrations (CC_50_) of Si/Cu (gel) and Si/Cu/500 nanoparticles in MDCK (**a**), A549 (**b**), CCL-1 (**c**), and BJ (**d**) cell cultures. Each point represents the percentage of cell viability as compared with the untreated cell control, which is considered 100%. Statistical analysis—SD from two independent experiments.

Cytotoxicity was tested in two epithelial and one fibroblast cell lines (transformed) and one human normal non-tumor fibroblast (BJ) cell culture. All results are demonstrated in Table 1 and Figure 11. The morphological alternations of the cellular structure can be seen in Figure S1a–d. The microscopic visualization revealed crystals deposited at high concentrations due to the incomplete solubility of the particles in the aqueous medium. Cytotoxicity was mostly of the round-cell degeneration type, and cell death resulted in reduced cell viability and optical density recorded. The highest toxicity was detected in MDCK cells, as indicated by the lowest CC_50_ of 50.19 and 53.01 μg/mL, followed by A549, BJ (non-transformed, diploid), and CCL-1. In general, both types of nanoparticles showed comparable cytotoxicity, except Si/Cu/500 in CCL-1 with a CC_50_ highest value of 306.7 ± 0.32 μg/mL, i.e., lowest toxicity, as this result is to be further clarified. Additionally, epithelial cells appear more sensitive than fibroblasts, probably due to their metabolic activity and proliferation rates leading to greater accumulation of the substances.

### 2.5. Daphnia Magna Eco-Safety Test

Cladocera are common inhabitants of freshwater. They play a crucial role in the aquatic food chain. *Daphnia magna* is among the most common inhabitants of lakes, permanent pools, and temporary reservoirs. Under laboratory conditions, the optimal environment for the development of *D. magna* can be created and maintained continuously, which allows various experiments to be conducted. *D. magna* is the most commonly used invertebrate in testing standard acute and chronic aquatic toxicity tests for screening and risk assessment of the use of various substances in the environment. Its small size makes it suitable for testing the ecotoxicity of various substances. Of great importance are the different nanoparticles used in many industries and released into the aquatic environment for various reasons. This study’s objective was to expose *Daphnia magna* to different nanocomposites to perform acute toxicity studies. The copper-based nanoparticles are widely used in various applications, and their health effects on the aquatic environment are of great concern. There is not much research on their safety in comparison to water volumes and aquatic species. It has been found that copper concentrations as low as 8.4 µg L^−1^ can induce acute toxicity in *D. magna*, while 20.2 µg L^−1^ can cause chronic toxicity [37].

On the other hand, Si-based nanoformulations are increasingly being applied in both consumer products and biomedical applications, resulting in significantly higher exposure to humans and the environment, which may lead to negative health effects [22,23].

**Figure 12:**
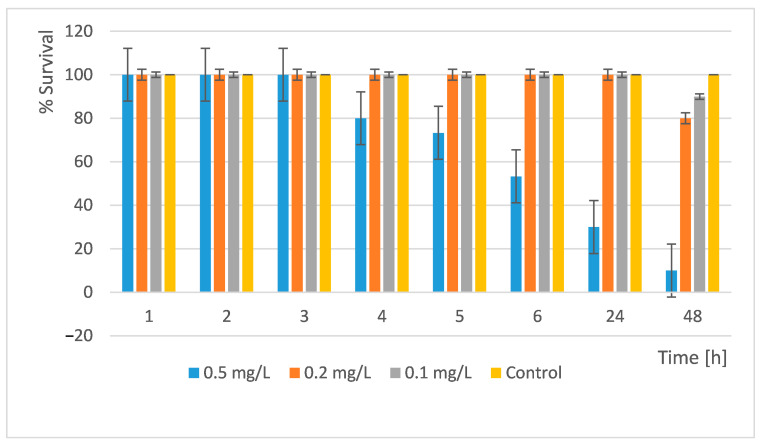
Survival rate of *Daphnia magna* after treatment with Si/Cu (gel).

**Figure 13:**
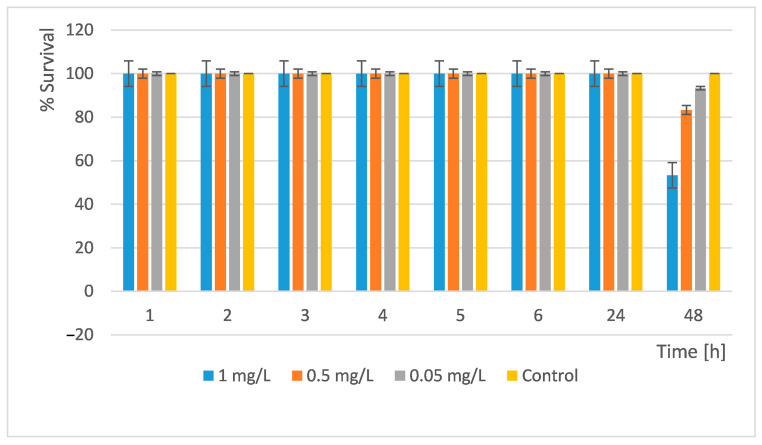
Survival rate of *Daphnia magna* after treatment with Si/Cu/500.

In our acute toxicity test, we treated *D. magna* with the newly synthesized nanocomposite materials and monitored their development and survival. We performed the readings at the 1st, 2nd, 3rd, 4th, 5th, 6th, 24th, and 48th h. The results obtained are shown in Figure 12 and Figure 13.

The results showed that a concentration of 0.5 mg/L is very toxic. Only 10% of all organisms survived within 48 h. At concentrations of 0.2 mg/L and 0.1 mg/L, the survival rate is high and similar. In the 0.2 mg/L, 80% survived and in the 0.1 mg/L, 90% of all daphnia survived (Figure 12). Therefore, at these concentrations, the substance used can be discharged into surface waters without harm to these organisms.

An acute toxicity test of the Si/Cu/500 nanocomposite was also performed. The results are presented in Figure 13.

The results showed that at concentrations of 0.05 mg/L, more than 93% of all daphnia survived. Thus, at these concentrations, the substance can be released into surface waters without causing harm to these scale organisms. The study showed that at a concentration of 1 mg/L the LC_50_ was reached. Si/Cu (gel) was more toxic to *D. magna* than the heat-treated material Si/Cu/500 (Figure 13).

The high toxicity of the Si/Cu/500 materials observed in our studies may be explained by the use of low-mineralized water. The effect of water hardness on copper toxicity in *D. magna* has been investigated through gene expression analysis, which demonstrates that exposure of *D. magna* to Cu in water with higher hardness levels led to a decrease in acute toxicity [38].

### 2.6. Mechanistic Hypotheses for Differences Between Si/Cu (Gel) and Si/Cu/500

Bactericidal effects: XRD and SEM results show that heat treatment at 500 °C increases crystallinity and grain size, leading to more ordered and reactive surface structures. This structural refinement likely improves the surface interactions with the bacterial membranes, explaining the higher bactericidal effect of Si/Cu/500. SEM images indicate that Si/Cu/500 has a rougher, more agglomerated surface, which may enhance physical interactions with bacteria (mechanical stress) but reduce dispersibility and effectiveness against larger eukaryotic cells such as yeasts.

Antifungal activity: EDS analysis shows a significant drop in surface copper content in Si/Cu/500 due to the diffusion of Cu into the silica matrix and oxidation during the annealing process. Since yeasts are more resistant and require higher surface availability of Cu^2+^ ions for effective inhibition, this could explain why Si/Cu (gel), with higher surface copper, was more effective against yeasts.

Ecotoxicity: The lower toxicity of Si/Cu/500 to *Daphnia magna* suggests that heat treatment also stabilizes the copper, reducing its bioavailability in aquatic systems—likely due to stronger Cu-O-Si bonding and reduced leaching.

Cytotoxicity: The lower toxicity of Si/Cu/500 to non-tumor cultures compared with Si/Cu (gel) could be due to lower Cu ion release, again due to deeper Cu embedding in the matrix. However, both materials remain cytotoxic at higher concentrations, probably due to accumulation-driven oxidative stress.

Oxidative activity: Chemiluminescence data show that both materials strongly inhibit ROS formation. This can be attributed to strong Cu incorporation into the silica matrix, limiting free Cu^2+^ release, thus preventing Fenton-type and superoxide-driven pro-oxidant reactions. This results in controlled ROS generation, which is beneficial in biological environments to reduce oxidative stress.

Some limitations of these nanocomposites in real-world applications could be cytotoxicity at higher concentrations, incomplete solubility and agglomeration, pH-sensitive activity, environmental accumulation, technically and economically challenging production, and bioaccumulation.

## 3. Materials and Methods

### 3.1. Used Materials and Preparation of the Gels

The gel with nominal composition 95SiO_2_/5CuO (mol%) was prepared using a sol–gel technique. A combination of the following precursors was applied for the synthesis: tetraethyl orthosilane (TEOS)—Sigma Aldrich Chemical (Burlington, MA, USA), Cu(OH)_2_, Fluka Chemie AG (Buchs, Switzerland), and C_2_H_5_OH (Sigma Aldrich). For the preparation of the solutions, silicon alkoxide was added under stirring in a mixture of 100 mL of absolute ethanol, where the appropriate amount of Cu(OH)_2_, was previously dissolved. To this solution, the stoichiometric amount of water (molar ratio H_2_O/TEOS = 1/4) for hydrolysis and polycondensation of the Si-(OEt) groups was added. A few drops of HCl were added to adjust the pH to a value of 3. The gelation occurred at room temperature, and it took about 10 h. The resulting homogeneous mixture was heated at 500 °C for 3 h. The samples obtained after heating had a dark color. The investigated samples were denoted as Si/Cu (gel) and Si/Cu/500 (500 °C heat-treated sample).

A schematic representation of the process can be found in Figure 13 in A. Bachvarova-Nedelcheva et al., 2004 [39].

### 3.2. Sample Characterization

Powder XRD patterns were registered at room temperature with a Bruker D8 Advance (Berlin, Germany) X-ray powder diffractometer with Cu Ka radiation (k = 1.54056 Å) with a LynxEye solid position-sensitive detector and an X-ray tube operating at 40 kV and 40 mA. X-ray diffraction patterns were recorded in the range of 5.3–80° 2 h with a step of 0.02° 2 h. The morphology and qualitative elemental analysis were studied by JEOL JSM 6390 (Tokyo, Japan). The SEM images were recorded with detectors for secondary and backscattered electrons. The accelerating voltage was 20 kV. The EDS has a 10 mm^2^ detector with 140 eV resolution and is produced by Oxford (INCA Oxford Instruments, Oxford, UK). The samples were studied as powders. Prior to the analysis, the surface of the samples was coated with a thin layer of gold.

### 3.3. Materials Used for the Antimicrobial Activity Testing

The National Bank for Industrial Microorganisms and Cell Cultures (NBIMCC, Sofia, Bulgaria) supplied the microorganisms employed in this investigation. Two types of Gram-positive bacteria were used, *Staphylococcus aureus* ATCC 25923 and *Bacillus cereus* ATCC 11778, along with three Gram-negative bacteria—*Salmonella enterica* ATCC 14028, *Escherichia coli* ATCC25922, and *Pseudomonas aeruginosa* ATCC 27853. Additionally, *Saccharomyces cerevisiae* CCY 21-6-3 and *Candida albicans* ATCC 18804, commensal eukaryotic microorganisms, were employed. The experiment started with preparing a fresh bacterial culture from a stock grown overnight on an agar plate. The bacteria were subcultured in appropriate broth to obtain a mid-log phase growth. The bacterial suspension was standardized using the McFarland turbidity standard. The suspension was adjusted to reach a 0.5 McFarland standard, equivalent to approximately 1–2 × 10^8^ CFU/mL. The nanoparticles were sonicated for 1 h in a Sonoplus (Berlin, Germany), and serial dilutions in a suitable solvent were prepared, ensuring that the concentration range encompassed those reported to have antibacterial activity and ensuring proper dispersion to prevent aggregation of nanoparticles, which could affect the results. In a 96-well microtiter plate, 100 µL of Mueller–Hinton broth was added to each well, preparing serial dilutions of the antimicrobial agent in the optimum range. To ensure even inoculum distribution, 100 µL of the standardized bacterial solution was applied to each well. The final bacterial concentration should be approximately 5 × 10^7^ CFU/mL in each well. For 18 to 24 h, the microtiter plate was incubated at 37 °C. Following incubation, the MIC and MBC were recorded, and the wells were checked for obvious growth.

### 3.4. Methodology for Evaluation of the Minimum Inhibitory (MIC) and Minimal Bactericidal Concentration (MBC) and Antibacterial Mode of the Si/Cu Nanocomposites

The determination of the MIC and MBC is essential for assessing the antimicrobial activity of antibiotics or nanoparticles. The MBC is the lowest concentration of an antimicrobial agent that kills a specific bacterium, while the MIC is the lowest concentration that inhibits visible bacterial growth. The antibacterial mode of inhibition refers to the mechanism by which these agents exert their bacteriostatic or bactericidal effects. After incubation, the colonies were counted. As a negative control, an antimicrobial agent was absent from the bacterial inoculum. Three independent replicates of the experiments were conducted, and the average of the values from each replication was calculated. The data are presented as the mean ± SD from three independent replicates (*n* = 3).

### 3.5. Chemiluminescent Assay

Chemiluminescence is a method that enables the monitoring of the concentration and kinetics of free radicals and ROS generation using small sample volumes (480–580 nm). This method allows for the dynamic observation of free radical reactions and the determination of their pro-oxidant/anti-oxidant (inhibitory) activity, thereby assessing the harmful or beneficial effects of the studied sample. The inherently weak signal in these reactions can be significantly amplified with the use of physical and chemical activators (probes). Automated computer-based registration during these studies allows for the real-time monitoring of reaction kinetics, quantum yields, the determination of rate constants, and other parameters during interaction with the tested substances.

A final active concentration of 1 mg/mL was used, which is considered significantly high. The chemiluminescent signal was compared to that of a control reaction without the presence of nanomaterials (blank control). The effect of the newly synthesized materials on the kinetics of free radical oxidation reactions was investigated in buffered ex vivo systems under various reaction conditions (physiological pH 7.4 and pH 8.5—favoring radical generation reactions, 25 °C), using the activated by lucigenin chemiluminescence method in the following model systems:

- Fenton’s system (H_2_O_2_–FeSO_4_)—for the generation of hydroxyl (·OH) and hydroperoxyl (·OOH) radicals;
- System with hydrogen peroxide (H_2_O_2_);
- (NADH–phenazine methosulfate) system, for the generation of superoxide radicals (O_2_^−^·).

All obtained data were statistically processed, and significant effects were expressed as quantum yields—an integral value and calculation, representing the observed effects.

- Fenton’s system: The sample contained 0.2 mol sodium hydrogen phosphate buffer with the chosen pH, Fenton’s reagent: FeSO_4_ (5 × 10^−4^ mol)—H_2_O_2_ (1.5%), and a chemiluminescent probe, lucigenin (10^−4^ mol). Free radicals and ROS were generated in the system according to the following scheme:
  (1) Fe^2+^ + H_2_O_2_ → Fe^3+^ + ·OH+ ^−^OH
  (2) Fe^3+^ + H_2_O_2_ → Fe^2+^ + ·OOH + H^+^
- System with hydrogen peroxide (H_2_O_2_): The sample contained 0.2 mol sodium hydrogen phosphate buffer with the chosen pH, H_2_O_2_ (1.5%), and a chemiluminescent probe, lucigenin (10^−4^ mol). In this system, hydrogen peroxide acts as both an oxidant and an ROS.
- NADH–phenazine methosulfate system: The sample contained 0.2 mol sodium hydrogen phosphate buffer with the chosen pH, NADH (10^−4^ mol), phenazine methosulfate (10^−6^ mol), and a chemiluminescent probe, lucigenin (10^−4^ mol). The scheme for superoxide radical generation in this chemical system is as follows:
  (1) PhMS + NAD.H + H^+^ → PhMS.H_2_ + NAD^+^
  (2) PhMS.H_2_ + PhMS → 2 PhMS.H^.^
  (3) PhMS.H^.^ + O_2_ → PhMS + O_2_^−.^ + H^+^

The reactions were monitored for 3 min, every 3 s. All tested materials were sonicated for 60 min before application. All experiments were performed in triplicates, *p* ≤ 0.05.

### 3.6. Cytotoxicity

#### 3.6.1. Compounds

Si/Cu (gel) and Si/Cu/500 nanoparticles were provided for analysis as a dry crystalline substance with a black color. The substances were dissolved in sterile conditions ex tempore at the beginning of each experiment in MEM or Dulbecco MEM (DMEM) maintenance medium, the stock solutions being properly homogenized with intensive resuspension and ultrasound for 15 min before the application at working concentrations varying between 1 and 1000 μg/mL.

#### 3.6.2. Cell Cultures

Madine–Darby canine kidney—MDCK (ATCC-CCL-34™), human lung carcinoma A549 (CCL-185™), Calu-3 (HTB-55™), mouse fibroblast cell lines (ATCC-CCL-1™, NCTC clone 929), and human normal dermal BJ (ATCC-CRL-2522™) were cultured at 37 °C in a 5% CO_2_ incubator Thermo Forma 310 (Thermo Fisher Scientific, Waltham, MA, USA), as monolayer cultures in T75 polystyrene flasks and 96-well plates (Corning^®^ Costar^®^, Corning, NY, USA) in MEM (CCL-1) and DMEM growth medium (Gibco, Grand Island, NY, USA) containing 10% fetal calf or horse serum (Gibco), 3.7 mg/mL sodium bicarbonate, 10 µM HEPES buffer (AppliChem GmbH, Darmstadt, Germany), 100 U/mL Penicillin, 100 µg/mL Streptomycin, and 25 µg/mL Amphotericin B. After thawing, cells in the mattresses were passaged every 72 h upon reaching 80–90% confluence by treatment with trypsin-EDTA solution (0.05% trypsin, 0.53 mmol EDTA). They were counted in a Burker chamber and resuspended in fresh growth medium. Cytotoxicity experiments were performed in 24 h cell cultures at a density of 2.5 × 10^5^/mL, at a minimum of 90% confluency of the monolayer, with visual and colorimetric readings performed at 48 h after treatment.

#### 3.6.3. Cytotoxicity Evaluation

After treatment of the cell monolayer with each of the agents in a respective dilution (including controls with no compounds), the 96-well plates were incubated at 37 °C and 5% CO_2_ for 48 h. The treatment effect was first assessed visually under a light inverted microscope (Olympus CK 40, Tokyo, Japan), photographed at ×10 magnification, and then assessed by staining with NR dye, as described previously [40]. The absorbance of light by the samples was measured at 540 nm using a microplate reader (Biotek Organon, West Chester, PA, USA).

Toxicity was calculated as a percentage of untreated cell controls, and 50% cytotoxic concentration (CC_50_) was determined as the concentration of the substance that causes 50% damage to cells after treatment by the formula:

Cell viability % = [OD toxicity sample/OD cell control] × 100,(1)

where OD stands for optical density.

#### 3.6.4. Statistical Analysis of the Cytotoxicity Evaluations

The tests were conducted in duplicate with four wells being treated with each concentration within the experiment for statistical reliability of the results. The data were processed using Gen5^®^ Microplate Reader and Imager Software 3.x and Excel^®^ MS Office 2021 software, and their graphical presentation was performed with Origin 8.5^®^.

### 3.7. Daphnia Magna Toxicity Test

The test was conducted according to the acute lethality toxicity protocol OECD (2004) and guideline for the testing chemicals acute immobilization test (2012) Test No: 211. The experiments used 3 concentrations of the nanoparticles—0.5 mg/L, 0.2 mg/L, and 0.1 mg/mL, with 3 replicates of each concentration, including 2 controls. The experiments were conducted by selecting 10 specimens of newborn small daphnia and placing them in 250 mL beakers filled with 100 mL of standing water, to which we added the test substance.

### 3.8. Statistical Analysis

The obtained results are statistically reliable and calculated by an independent *t*-test, *p* ≤ 0.05. All data were processed using MSOffice Pro 2021 and Origin Pro 8, unless mentioned otherwise.

## 4. Conclusions

Si/Cu-hybrid materials have been prepared by applying a sol–gel method. The phase analysis showed that all samples are characterized by well-defined diffraction peaks corresponding to CuO. Their biological effects can be reviewed as follows:

- Most bacteria were more resistant to the Si/Cu (gel) material than to the heat-treated nanoparticles Si/Cu/500. The opposite effect was observed on yeasts—they were more sensitive to the Si/Cu (gel) nanoparticles and more resistant to the heat-treated Si/Cu/500 ones. The observed resistance could be explained by both the structure of the cell wall and the content of the hybrid material.
- Cytotoxic effects observed were mostly of the round-cell degeneration type, leading to cell death. The epithelial tumor cells appeared to be more sensitive than the fibroblasts from a non-tumor origin, probably due to their metabolic activity and proliferation rates leading to greater accumulation of the substances.
- The results from the luminescent study showed that the tested nanomaterials significantly inhibited the generation of ROS in different model systems, strongly influenced by the pH of the media. This underlines the need for careful material selection based on the needed application as well as the potential of these new nanohybrids as regulators and inhibitors of the free radical processes in the living systems. The antimicrobial and modeled oxidation tests support the application of the new nanohybrids in living, eukaryotic systems.
- Cytotoxicity results showing higher tolerance of normal, non-transformed than tumor cells suggest that these nanomaterials are safe when used at appropriate concentrations and conditions.

The ecotoxicity study using *Daphnia magna* revealed that the heat-treated Si/Cu/500 nanohybrid exhibited significant toxicity, reaching the median lethal concentration at lower exposures. Si/Cu (gel) was more toxic to *D. magna* than the heat-treated material (Si/Cu/500).

The obtained data suggest that the as-prepared nanohybrids could be used in various biotechnological implementations.

## Figures and Tables

**Table 1:** Cytotoxicity evaluation in MDCK, A549, CCL-1, and BJ cell cultures (statistical analysis: SD *—standard deviation from two independent experiments).

Compound	CYTOTOXICITY CC_50_ ± SD * (μg/mL)			
	Cell Culture			
	MDCK	A549	CCL-1	BJ
Si/Cu (gel)	50.19 ± 2.09	61.66 ± 0.61	87.8 ±1.83	75.7 ± 0.04
Si/Cu/500	53.01 ± 2.67	50.48 ± 0.17	306.7 ± 0.32	71.22 ± 0.34

## Data Availability

Data is contained in the paper.

## Supplementary Materials

The following supporting information can be downloaded at: https://www.mdpi.com/article/10.3390/ph18070976/s1, Figure S1. Morphological evaluations of Si/Cu (gel) and Si/Cu/500 nanoparticles toxicity in MDCK (a), A549 (b), CCL-1 (c), and BJ (d) cell lines under an inverted light microscope Olympus CK40 (Tokyo, Japan), magnification 10×.
