# Supplementary material for: Antimicrobial, Oxidant, Cytotoxic, and Eco-Safety Properties of Sol–Gel-Prepared Silica–Copper Nanocomposite Materials
Source: Pharmaceuticals (Basel). 2025 Jun 28;18(7):976. doi: 10.3390/ph18070976 (PMC12299540; doi:10.3390/ph18070976)
Supplement: Supplementary file 1 [file pharmaceuticals-18-00976-s001.zip › pharmaceuticals-3673561-supplementary.pdf]

Antimicrobial, Oxidant, Cytotoxic and Eco-safety Properties of Sol-Gel Prepared Silica-Copper Nanocomposite Materials
by L. Yordanova et al., 2025

Supplement S1

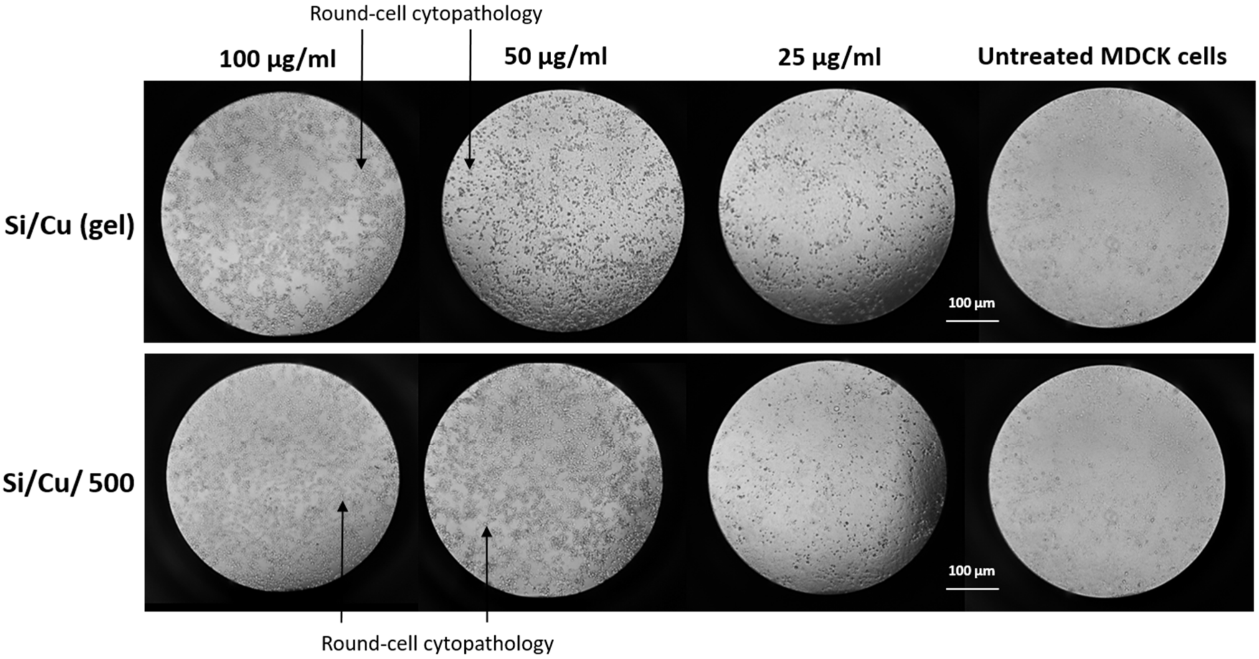

(a)

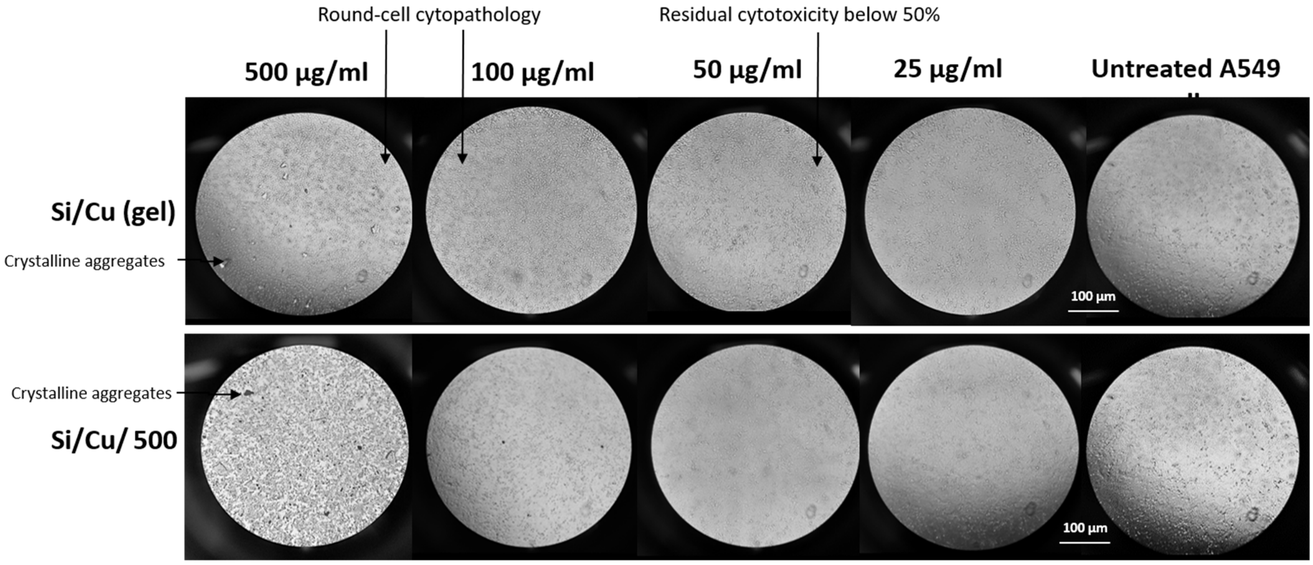

(b)

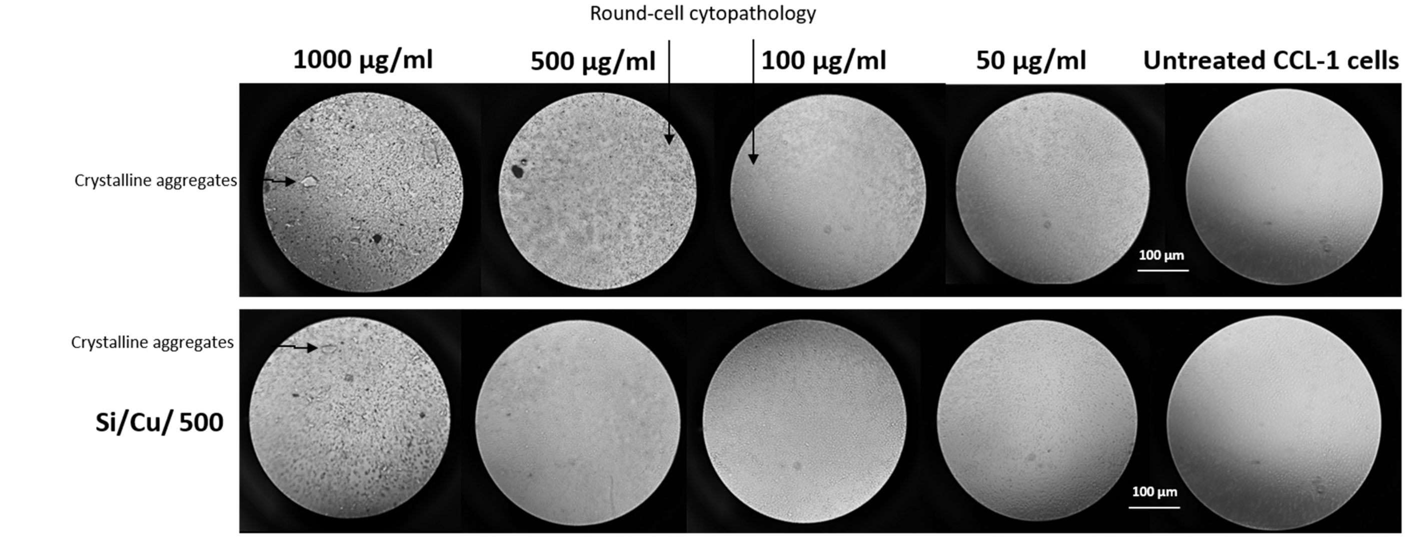

(c)

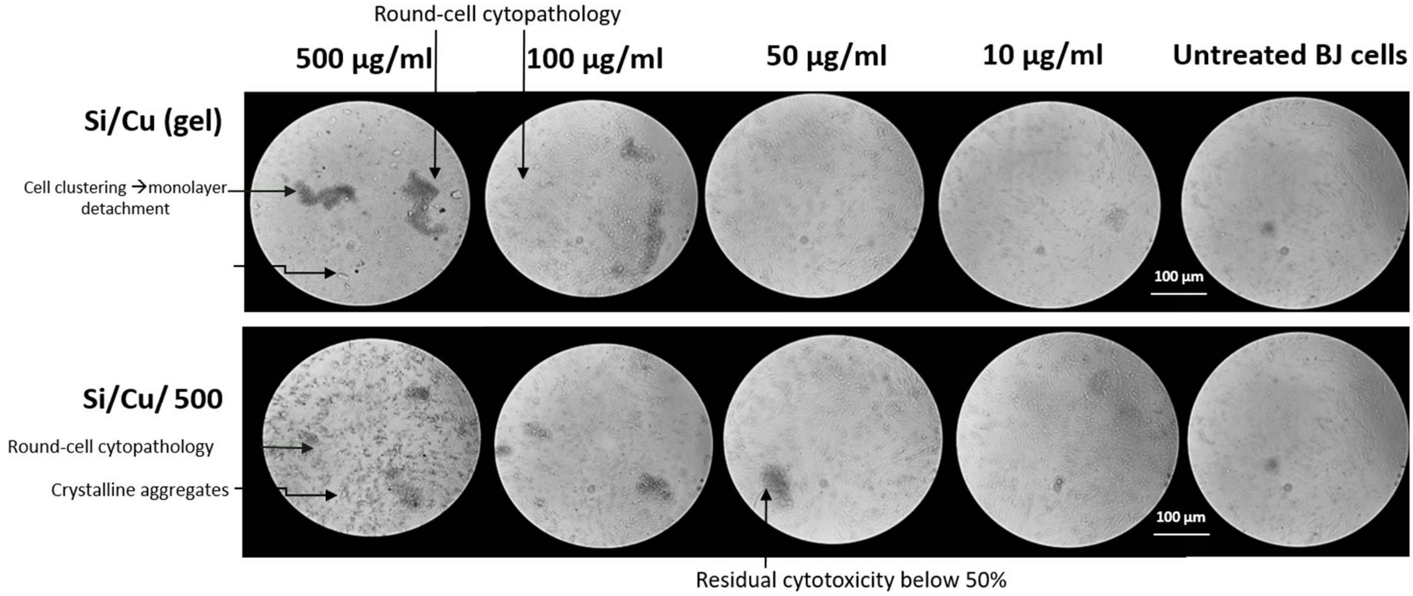

(d)

Figure S1. Morphological evaluations of Si/Cu (gel) and Si/Cu/500 nanoparticles toxicity in MDCK (a), A549 (b), CCL-1 (c), and BJ (d) cell lines under an inverted light microscope Olympus CK40, magnification 10X.
